# Butyrylcholinesterase level in poisoned patients with phosphide compounds

**DOI:** 10.22088/cjim.10.4.458

**Published:** 2019

**Authors:** Saeed Afzali, Seyed Kazem Taheri, Mohamadali Seifrabiei

**Affiliations:** 1 Department of Forensic Medicine and Toxicology, School of Medicine, Hamadan University of Medical Sciences, Hamadan, Iran; 2 Department of Community Medicine, School of Medicine, Hamadan University of Medical Sciences, Hamadan, Iran

**Keywords:** Zinc Phosphide, Aluminum Phosphide, Butyrylcholinesterase.

## Abstract

**Background:**

Metal phosphides are widely used as a rodenticide and insecticide and poisoning with these substances has a very high mortality. The aim of this study was to evaluate the butyrylcholinesterase (BuCh) level in poisoning with metal phosphides.

**Methods:**

In this case series study, 60 poisoned patients with zinc phosphide (ZnP) and aluminum phosphide (ALP) who was admitted to poisoning ward in Hamadan, Iran, enrolled in the study and BuCh level was measured. The sex, age, amount of ingestion, time of consumption, signs and symptoms, ECG and arterial blood gas (ABG) changes and clinical outcomes were evaluated.

**Results:**

Most (58.3%) patients were males, the mean age was 32.76±17.65 years. The average amount of ingestion was 7.5±4.6 and 2.5±2.96 gr for ZnP and ALP, respectively. Most signs and symptoms were hypotension (97%), nausea and vomiting (82%) and abdominal pain (32%). The average amount of BuCh level in all patients was 5163.25±2158.31 U/L, that's while in ZnP and ALP poisoned patients was 5608±1577 U/L and 4721.46±2562U/L respectively. The most dysrhythmia (26.7%) was sinus tachycardia. Acidosis was seen in 33.3% of patients. 14 patients died after hospital admission, which caused a 23.3% fatality rate.

**Conclusion:**

Our results showed that, there was no significant change in BuCh level in poisoned patients with phosphide compounds.

Today, the use of pesticides in the world is considered as a recognized health problem. It is estimated that around 30% of global suicides are due to pesticide self-poisoning (1). Metal phosphides pesticides are highly toxic, low cost, and are easily accessible as rodenticide agents and used for self-poisoning (2). Zinc phosphide (ZnP) and aluminum phosphide (ALP) are among the most important metal phosphides. Poisoning with these agents is frequently seen in India, Srilanka and Iran (3-8). In a recent study, the incidence of fatal aluminum phosphide poisoning cases referred for phosphine analysis was 5.22 and 37.02 per million of population of Tehran, Iran in 2006 and 2013, respectively (9).The mechanism of toxicity is not clearly defined. ZnP and ALP toxicity is related to the production of phosphine gas. Phosphine inhibits cytochrome c oxidase and causes mitochondrial destruction and inhibits oxidative respiration which leads to anaerobic metabolism, severe metabolic acidosis, multi-organ dysfunction and death (10-13). Phosphine can lead to severe cardiac suppression and cardiogenic shock (14). One of the proposed mechanisms of toxicity is the reduction of the true cholinesterase and serum cholinesterase (butyrylcholinesterase) enzyme, but usually do not show obvious clinical manifestations (15-17).

According to the few human studies on this subject in the world and particularly in Iran, and considering that it was not possible for us to measure true cholinesterase enzyme level, this study aimed to measure the butyrylcholinesterase (BuCh) level in human poisoned patients by ZnP and ALP. We assessed symptoms and signs, ECG and ABG changes, liver and kidney function tests and outcome between ZnP and ALP poisoned patient groups. 

## Methods

**Study design: **This was a case series study and all poisoned patients with metal phosphides include ZnP and ALP was investigated. This study was conducted in a 12-month period from December 2016 to December 2017 and all of the poisoned patients with ZnP or ALP who referred to the hospital during the study period were enrolled in the study. Totally 60 patients, including 30 ZnP and 30 ALP poisoned patients were investigated.

**Setting: **The study was carried out at Farshchian Hospital in Hamadan (west Iran). This hospital is a poison center that covers about 1,500,000 people in the Hamadan province. This is the only tertiary hospital for the poisoned patients in this province. 

**Data collection: **The diagnosis of ZnP and ALP consumption were determined based on the history, clinical signs and symptoms and paraclinical tests such as SGOT, SGPT, BUN, creatinine, arterial blood gas (ABG), ECG were also checked out. In addition, BuCh was measured by BuCh Elisa kits of Pars Azmoon Company. The reference range of this kit was 3930–11500 U/L. Demographic data, clinical and paraclinical signs, BuCh level and outcome of poisoning were compared between ZnP and ALP poisoned patients. Those patients who consumed ZnP and ALP lower than toxic dosage and all patients who had one of the factors affecting BuCh level, such as consumption of organophosphates or carbamates, pregnancy and chronic liver diseases were excluded. The data were entered into the SPSS statistical software, Version 21. The analysis was mostly descriptive in nature; differences between two groups were evaluated with the unpaired student’s t-test and chi-square test. Of all comparisons, a p-value less than 0.05 was considered significant.

## Results

 In 60 poisoned patients by phosphide compounds 35 (58.3%) were males and 25 (41.7%) were females. The mean age of the poisoned patients was 32.76±17.65 years (30.46±19.82 years in ZnP and 35.06±15.16 years in ALP, respectively). Most patients (50 patients or 83%) had ingested phosphide compounds less than 6 hours before referring to the emergency ward and for the rest 10 patients (17%) the referral time was more than 6 hours. 14 out of 60 poisoned patients died after hospital admission, which showed a 23.3% fatality rate among patients. The demographic data, time of ingestion and final outcome in ZnP and ALP poisoned patients are presented in table 1.

**Table 1:** Demographic characteristics of 60 hospitalized patients with phosphide compounds consumption

**Variables**	ZnP* **(N%)**	ALP** **(N%)**	**Pvalue**
Sex			
Male Female	14 (46.7) 16 (53.3)	21(70) 9(30)	0.06
Age (y)			
10-20 21-30 31-40 41-50 >50	11(36.7) 11(36.7) 3(10) 1(3.3) 4(13.3)	6(20) 7(23.3) 7(23.3) 4(13.3) 6(20)	0.32
Time of ingestion (h)			
<2 2-6 >6	12(40) 11(36.7) 7(23.3)	13(43.3) 14(46.7) 3(10)	0.37
Fatality rate	4(13.3)	10(33.3)	0.06

* Zine phosphide

** Aluminium phosphide

The average ingestion of ZnP was 7.5±4.68 gr. 23 (76.7%) patients had ingested 5 gr, 2 (6.7%) patients ingested 10 gr and 5 (16.6%) patients had more than 10 gr. The average ingestion of the ALP was 2.5±2.96 gr; from them 13 (43.3%) patients ingested 0.5 gr, 4 (13.3%) patients consumed ate 1.5 gr, 8 (26.7%) patients had 3 gr and 5 (16.7%) patients ingested more than 3 gr. 

The most frequent symptoms in 60 poisoned patients were: hypotension (58 patients, 97%), nausea and vomiting (49 patients, 82%), loss of conscious (21 patients, 35%), abdominal pain (19 patients, 32%), tachypnea and shock (each of the 14 patients, 23%) and agitation (7 patients, 12%). According to ECG disturbances, in all 60 poisoned patients, 50% (30 people) had normal sinus rhythm and in another 30 patients, frequency of dysrhythmia was as follows: 16 (26.7%) patients had sinus tachycardia, 11 (18.3%) patients had ventricular tachycardia and 3 (5%) patients had supra ventricular tachycardia. According to ABG analysis acidosis was seen in 20 patients (33.3%) patients.

In a total of 60 patients, the average amount of BuCh level was 5163.25±2158.31 U/L. The minimum and maximum level of BuCh in the patients was 435 U/L and 10076 U/L. In ZnP and ALP poisoned patients, the mean level of Buch was 5608±1577 U/L and 4721.46±2562U/L respectively (graph1). According to liver enzyme change (AST, ALT); only 1 (1.7%) patient had abnormal liver enzyme level.

**Figure 1:**
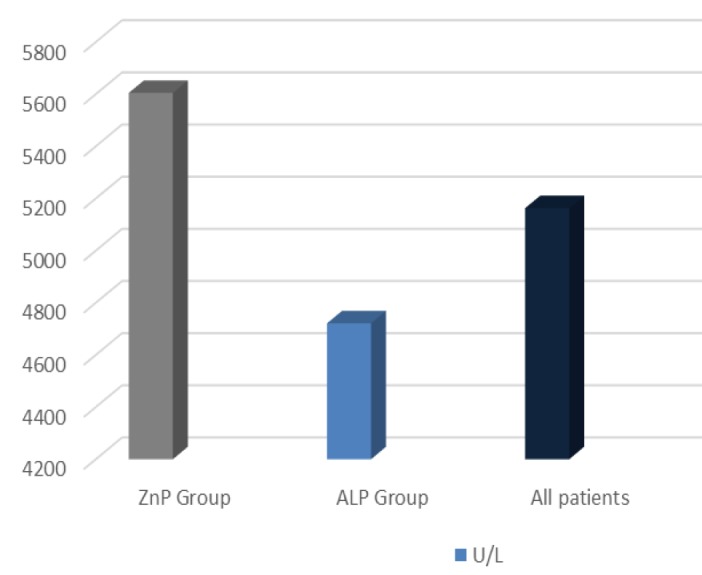
Mean butyrylcholinesterase level in the participants

## Discussion

Poisoning by pesticides is a common problem in developing countries. These properties are the best products for self- poisoning. Among them, poisoning with phosphide compounds such as ZnP and AlP is associated with a very high mortality. ZnP as a rodenticide is a gray-black powder with an odor similar to garlic. Ingestion of 1 gr of ZnP can cause toxicity in humans and death has been reported after ingestion of 4-8 gr (18). AlP exists as yellowish to dark gray granular or powdered solid which is formulated as tablets, pellets, or small sachets of powder and used as a rodenticide, insecticide, and fumigant for stored cereal grains. Each tablet contains 3 gr of ALP and ingestion of 1 – 1.5 gr can be lethal (19, 20). 

 In our study, the average consumption of the ALP was 2.5 gr and in Shadnia’s study was 5.1 gr (7). After exposure of these compounds to the air, moisture and gastric hydrochloric acid produce a highly toxic phosphine gas. Although the exact mechanism of phosphine toxicity is unknown but after rapidly absorbed throughout the gastrointestinal tract, leading to systemic toxic effects. The mechanism of toxicity includes failure of cellular respiration due to the effect on mitochondria, inhibition of cytochrome C oxidase and formation of highly reactive hydroxyl radicals. Cellular injury due to lipid peroxidation is also suggested. The exact underlying mechanism of cardiotoxicity and acute circulatory failure caused by phosphine is not well defined (20). There is a decrease in the level of catalase and increase in the activity of superoxide dismutase in patients of phosphine poisoning (21). Also, the reduction of glutathione concentration in different tissues explains the cellular injury as glutathione is a protecting factor against oxidation by catalysing the reduction of the oxygen peroxide in O2 and H2O (13).

 Phosphine, either during inhalation or exhalation after ingestion, directly produces injury to the alveolar capillary membrane, in addition to oxidative injury leading to acute lung injury (20). Reducing the BuCh enzyme is one of the other effects of phosphine gas (17). Based on Al-Azzawi and Mittra’s studies which were done on the rat and the human serum exposed to phosphine, BuCh level decreased (15-16). In our study, during a-12 month period, 60 patients who were admitted due to poisoning by ZnP and ALP were evaluated. The results showed that there was no significant change in the BuCh level in phosphide compounds respecting the normal level and only in a small number of patients, enzyme reduction was observed. Despite the decrease of this enzyme in a small number of patients, no cholinergic symptoms and signs were observed.

Self-poisoning with pesticides, especially phosphide compounds is one of the most common cases of poisoning among males and females. The prevalence varies between males and females in different parts of the world, as in the present study, the number of female ZnP poisoned patients were more than males, while in another study, male patients were more than females (9). Also, in our study, the number of ALP poisoned male patients was more than females, while in Shadnia’s study males and females were equal and in Soltaninejad’s study, males were more than females (7, 22). 

Suicide by pesticides, as in other cases of deliberate poisoning is more common in young adults (23). The average age of patients in the present study was 32.8 years and most of them were between 20-30 years and in Shadnia’s study was 27.1 years and were between 20-30 years (7). Due to the high toxicity of phosphide compounds, the mortality rate is very high and the most common cause of death usually is cardiac arrhythmias and cardiogenic shock (24). The mortality rate is highly variable, ranging from 37 to 100%, and can reach more than 60%, even inexperienced and well-equipped hospitals (25). 

In this study, the fatality rate in ZnP and ALP poisoned patients was 16.7% and 33.3%, respectively, whereas in Trakulsrichai’s study ZnP fatality was 7 % (20) while in Soltaninejad’s and Bhalla’s studies, ALP fatality was 57.5% and 40%, respectively (22, 26). The most clinical manifestations include vomiting, abdominal pain, agitation, tachy dysrhythmias, tachypnoea, acidosis, hypotension, palpitation and unresponsive shock (24). In most studies, similar to our study, cardiovascular and gastrointestinal symptoms were the most clinical manifestations (7, 9). About 50% of our patients had dysrhythmia which was similar to the Soltaninejad’s study (22). The most dysrhythmias in our study were sinus tachycardia, ventricular tachycardia, supraventricular tachycardia while in Jha’s study was sinus tachycardia and sinus bradycardia (27).

In this study, like the other studies, the most finding in ABG was acidosis and most patients who had severe acidosis died (7, 28). 

Considering that the present study was a case series study and carried out within a one – year period, a limited number of patients were assessed. On the other hand, regarding the fact that most of the studies measuring the cholinesterase enzyme have been performed on animal samples, comparison of the results of our study with other studies will be of limited value. Also, considering that it was not possible to measure the true cholinesterase enzyme of this medical center, it would be better to consider this issue in future studies.

In Due to lack of a specific antidote, poisoning with ZnP and ALP still causes fatalities. Cardiovascular and GI tract symptoms were the most important clinical manifestations. These symptoms were more frequent in ALP poisoned patients. Most patients who had decreased of BuCh enzyme were ALP poisoning patients. 
